# Effect of Storage Temperature on Structure and Function of Cultured Human Oral Keratinocytes

**DOI:** 10.1371/journal.pone.0128306

**Published:** 2015-06-08

**Authors:** Rakibul Islam, Catherine Jackson, Jon R. Eidet, Edward B. Messelt, Rima Maria Corraya, Torstein Lyberg, May Griffith, Darlene A. Dartt, Tor P. Utheim

**Affiliations:** 1 Department of Oral Biology, Faculty of Dentistry, University of Oslo, Oslo, Norway; 2 Department of Medical Biochemistry, Oslo University Hospital, Oslo, Norway; 3 Integrative Regenerative Medicine Centre, Department of Clinical and Experimental Medicine, Linköping University, Linköping, Sweden; 4 Schepens Eye Research Institute/Massachusetts Eye and Ear, Department of Ophthalmology, Harvard Medical School, Boston, MA, United States of America; Instituto Butantan, BRAZIL

## Abstract

**Purpose/Aims:**

To assess the effect of storage temperature on the viability, phenotype, metabolism, and morphology of cultured human oral keratinocytes (HOK).

**Materials and Methods:**

Cultured HOK cells were stored in HEPES- and sodium bicarbonate-buffered Minimum Essential Medium (MEM) at nine temperatures in approximately 4°C increments from 4°C to 37°C for seven days. Cells were characterized for viability by calcein fluorescence, phenotype retention by immunocytochemistry, metabolic parameters (pH, glucose, lactate, and O_2_) within the storage medium by blood gas analysis, and morphology by scanning electron microscopy and light microscopy.

**Results:**

Relative to the cultured, but non-stored control cells, a high percentage of viable cells were retained only in the 12°C and 16°C storage groups (85%±13% and 68%±10%, respectively). Expression of ABCG2, Bmi1, C/EBPδ, PCNA, cytokeratin 18, and caspase-3 were preserved after storage in the 5 groups between 4°C and 20°C, compared to the non-stored control. Glucose, pH and pO_2_ in the storage medium declined, whereas lactate increased with increasing storage temperature. Morphology was best preserved following storage of the three groups between 12°C, 16°C, and 20°C.

**Conclusion:**

We conclude that storage temperatures of 12°C and 16°C were optimal for maintenance of cell viability, phenotype, and morphology of cultured HOK. The storage method described in the present study may be applicable for other cell types and tissues; thus its significance may extend beyond HOK and the field of ophthalmology.

## Introduction

Epithelial stem cells located in the peripheral limbal region of the cornea are critical for the health of the cornea [1, 2]. However, these stem cells can be destroyed by external factors, such as chemical/thermal burns, severe infections, as well as by a number of diseases, including Stevens-Johnson syndrome and congenital disorders such as aniridia. As a consequence of limbal stem cell loss ingrowth of conjunctival epithelial cells and blood vessels onto the cornea may develop. This condition, entitled limbal stem cell deficiency (LSCD), may result in severe pain and ultimately blindness.

Limbal stem cell deficiency can be treated by transplantation of cultured human limbal epithelial cells (HLEC) [3]. However, in bilateral severe LSCD, which is more common than unilateral disease, autologous cell sources other than limbal cells offer an alternative treatment without the need for prolonged immunosuppression [4]. Transplantation of autologous human oral keratinocytes (HOK) has the benefit of circumventing immune rejection as well as the risk of transferring infectious diseases from allogeneic donors. In animal models, a variety of cell types have been tested for potential LSCD treatment [4]. To date, only cultured conjunctival epithelial cells [5] and HOK have been clinically evaluated for LSCD treatment [6] Among the autologous sources to treat LSCD, HOK have the advantage of being the most extensively studied with currently 20 clinical studies demonstrating their potential in treating LSCD. The effect of HOK has been shown even in patients with Stevens–Johnson syndrome, which affects both oral and ocular mucosa, including corneal limbal epithelial cells [7].

Strict regulatory demands [8, 9] are expected to force the development of large centralized culture laboratories. As a consequence, cells will need to be transported between the centralized culture laboratories and eye clinics worldwide [9]. Storage of the transplant for at least a few days prior to surgery provides time for phenotypic investigations [10] and sterility control, [11] which may potentially increase the clinical outcome and the safety of the procedure. Moreover, it enables a logistical window for planning of surgery [12]. An effective and convenient short-term storage method would fulfill these purposes, but so far, no such protocol is available for HOK.

Cryopreservation is the standard method for storing cells in suspension, but it is less successful for storing adherent stratified epithelial cells [13, 14]. The two main mechanisms of cell injury during freezing cell suspensions are intracellular ice-formation and increased solute concentration [15]. Considering the challenges that are inherent to cryopreservation, our goal was to develop an easily implementable and inexpensive short-term storage method for cultured HOK. Previous publications on storage of cultured epithelial cells [16–21] demonstrated that storage temperature profoundly affects the viability, phenotype, metabolism and morphology of these cells. We hypothesized that storage temperature will similarly affect cultured HOK and conducted the present study to define the optimal temperature for storage of HOK.

## Materials and Methods

First passage normal HOK, oral keratinocyte medium (OKM), oral keratinocyte growth supplement (OKGS) and penicillin/streptomycin solution (P/S) were purchased from ScienCell Research Laboratories (San Diego, CA). Nunclon Δ-surface multidishes, plastic coverslips, pipettes and sealing tapes were purchased from VWR International (West Chester, PA). The calcein-acetoxymethyl ester (CAM) and the minimum essential medium (MEM) were obtained from Invitrogen (Carlsbad, CA). Phosphate-buffered saline (PBS), 4-(2-hydroxyethyl)-1-piperazineethanesulfonic acid (HEPES), bovine serum albumin (BSA), sodium bicarbonate, propidium iodide (PI) solution, Tween 20 and 4',6-diamidino-2-phenylindole (DAPI) were all from Sigma-Aldrich (St. Louis, MO). Rabbit polyclonal anti-cleaved caspase-3 antibody (Asp 175) was supplied by Cell Signaling Technology (Danvers, MA), while mouse monoclonal anti-proliferating cell nuclear antigen antibody (PCNA; clone PC10) was purchased from DAKO (Glostrup, Denmark). Rat monoclonal anti-BCRP/ABCG2 antibody (BXP-53), mouse monoclonal anti-cytokeratin 18 (CK18) antibody, rabbit polyclonal anti-C/EBPδ antibody, mouse monoclonal anti-Bmi1 antibody (clone 1.T.21), FITC-conjugated goat anti-mouse IgG and Cy3-conjugated goat anti-rabbit IgG were purchased from Abcam (Cambridge, UK) (Table 1).

**Table 1:** List of the antibodies.

Antibody Name	Dilution
Anti-ATP-binding cassette sub-family G member 2 (ABCG2)	1:300
B lymphoma Mo-MLV insertion region 1 homolog (Bmi1)	1:1000
CCAAT/enhancer-binding protein delta (C/EBPδ)	1:500
Proliferating cell nuclear antigen (PCNA)	1:500
Cytokeratin 18 (CK18)	1:1000
Caspase-3	1:400

### Culture and Storage of Human Oral Keratinocytes

HOK (passages 4 and 5) were seeded (5000 cells/cm^2^) on either Nunclon Δ-surface 48-well plates or on coverslips. They were maintained in complete OKM (made by adding 5mL OKGS and 5mL P/S to 500mL OKM), in a 37°C humidified incubator with 5% CO_2_. The culture medium was changed every other day. Confluent cultures were obtained on day five, at which time OKM was removed and the cultures were rinsed with PBS before adding the storage medium. The storage medium consisted of 1.0mL MEM, 25mM HEPES, 600mg/L sodium bicarbonate and 50*μ*g/mL gentamycin (hereafter named MEM). The multi-well dishes were sealed with sealing tapes and the cultures were randomized for storage at nine temperatures (4°C, 8°C, 12°C, 16°C, 20°C, 24°C, 28°C, 32°C, and 37°C) for seven days. Previously described custom-built storage cabinets with a very small standard deviation for the set temperatures (±0.4°C) were used for regulating temperature during storage [19]. In addition, the temperature inside each storage container was monitored throughout all experiments. Cells cultured for five days, but not subjected to storage, served as controls in all experiments.

### Analysis of Cellular Viability

**Fig 1:**
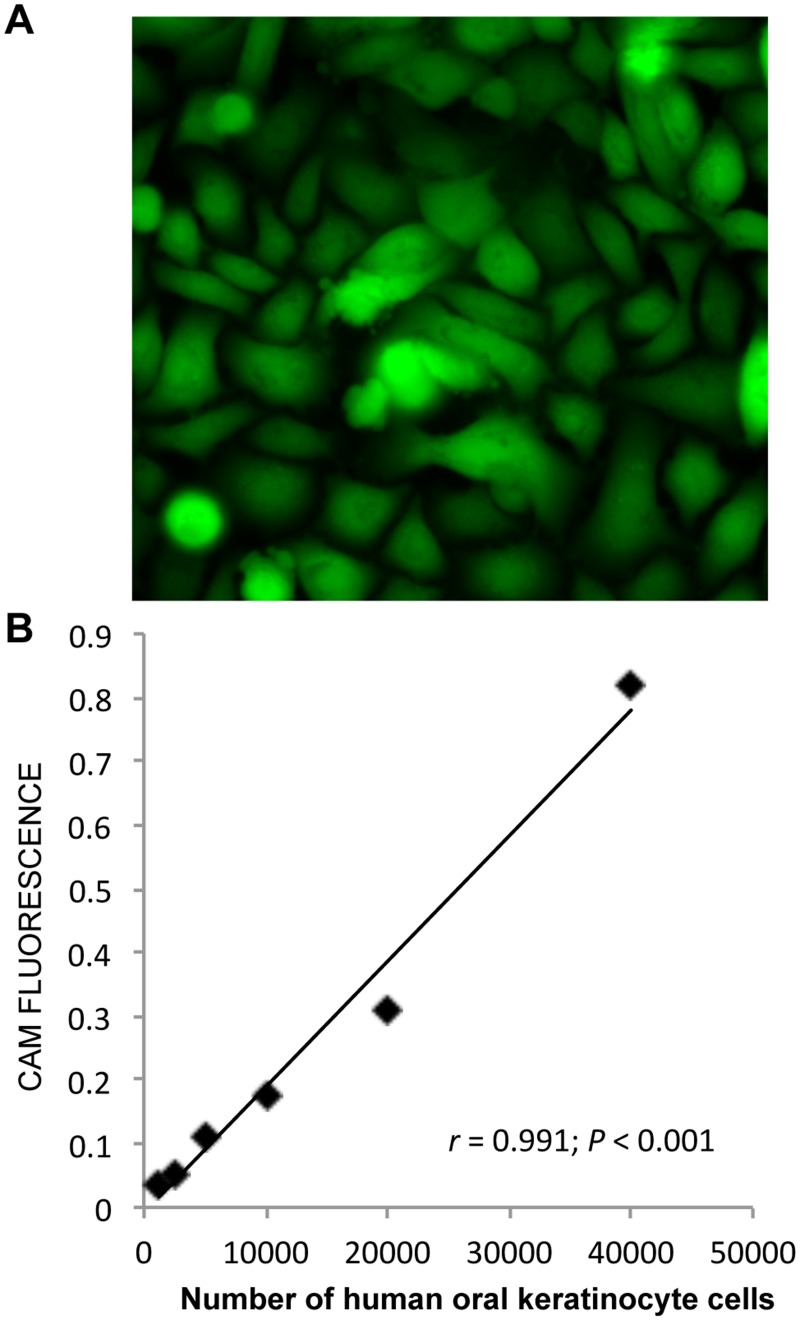
Calcein-acetoxymethyl ester (CAM), which exclusively stains live cells, was used to analyze cell survival. (A) Control cells were CAM positive (green). Original magnification: 200x. (B) HOK were seeded in multidishes at different concentrations and incubated for two hours to ensure attachment. The CAM reagent was added to the cells for one hour, and CAM fluorescence was measured with a microplate fluorometer (excitation/emission filter pair 485/538nm).

Cell viability was analyzed after one week of storage by incubating the cultures for one hour with PBS containing 1.0*μ*M acetomethoxy derivate of calcein (CAM) (1:2000) [14], which in living cells is enzymatically cleaved into the green fluorescent calcein (Fig 1A). Calcein fluorescence was quantified by a microplate fluorometer (Fluoroskan Ascent, Thermo Scientific, Waltham, MA) with the excitation/emission filter pair 485/538nm. Cultures were randomized to storage for one week at nine different temperatures, as described above. Cultures not subjected to storage served as control. The accuracy of this method was determined by adding increasing concentration of cells to multiwell dishes for 2 hours and then using CAM to determine viable cell. The amount of CAM fluorescence increased linearly with cell number (Fig 1A and 1B).

A standard curve of gradually increased concentration of seeded cells were prepared to demonstrate the reliability of the CAM measurements obtained by the microplate fluorometer (Fig 1B). Using a cell counter (Scepter 2.0 Cell Counter, Merck Millipore, Billerica, MA), cell suspensions with increasing cell concentrations were seeded in multidishes and left in the incubator for two hours to ensure cell attachment. The cells were then incubated with the CAM reagent as described above to stain the attached cells. The CAM fluorescence was thereafter measured by the microplate fluorometer. The number of seeded cells correlated highly with the measured CAM fluorescence, thereby showing great accuracy of the microplate fluorometer measurements (*r* = 0.991; *P* < 0.001) (Fig 1B).

### Immunocytochemical Analysis of Phenotype

Cells were cultured in 48-well plates and stored at the nine temperatures as described above. Samples were subsequently prepared for immunocytochemistry (ICC) by 10 minutes of methanol fixation at room temperature followed by 1 hour of permeabilization and blocking in PBS containing 1% BSA and 0.1% Tween 20. The samples were then incubated over night at 4°C with the antibodies that are listed in Table 1 diluted in blocking solution of PBS with 1% bovine serum albumin (BSA):

Replacing the primary antibody with PBS served as negative control. FITC-conjugated goat anti-mouse secondary antibodies (diluted 1:250 in blocking solution) and Cy3-conjugated goat anti-rabbit secondary antibodies (diluted 1:250) were added for one hour at room temperature. To counterstain cell nuclei 1*μ*g/mL DAPI was added to last wash solution. Photomicrographs were captured with a DS-Qil black-and-white camera at five random locations in each well using an epifluorescence microscope with a motorized microscope stage (Nikon Eclipse Ti; Nikon Instruments, Tokyo, Japan). Identical exposure time and gain were used for all compared groups, while keeping the image brightness within the camera’s dynamic range. ImageJ software (version 1.46r; http://rsbweb.nih.gov/ij) was used to convert the corresponding FITC/Cy3 and DAPI photomicrographs to 8-bit gray scale pictures. Semi-quantitative immunocytochemical analyses of the markers were carried out by counting the number of positively stained cells divided by the total number of cells in each image.

### Metabolic Analysis

Glucose, lactate, oxygen and pH measurements in the storage medium were performed by sampling 200μL of medium following one week of storage. The control solution consisted of freshly prepared storage medium. Each sample was run immediately in a Radiometer ABL 700 blood gas analyzer (Radiometer Copenhagen, Denmark).

### Scanning Electron Microscopic Analysis of Morphology

HOKs were cultured and stored as described above on coverslips and processed for scanning electron microscopy (SEM) as previously described [22]. In brief, stored cultures were fixed in 2.5% glutaraldehyde, dehydrated through an ethanol series, and critical point dried (Polaron E3 Critical Point Drier; Polaron Equipment Ltd, Watford, UK). The samples were then sputter-coated with a 30nm thick layer of platinum in a Polaron E5100 sputter coater, prior to examination under a XL30 EEM electron microscope (Philips, Amsterdam, Netherlands).

### Statistical Analysis

The one-way analysis of variance (ANOVA) with Tukey’s post hoc test (SPSS ver. 19.0) was used to compare the groups. Pearson´s correlation coefficient (*r*) was calculated for assessing the reliability of the microplate fluorometer measurements. Statistical significance was set at *P* ≤ 0.05. Data were expressed as mean ± standard error of the mean.

## Results

### Effect of Storage Temperature on Viability of Stored Human Oral Keratinocytes

**Fig 2:**
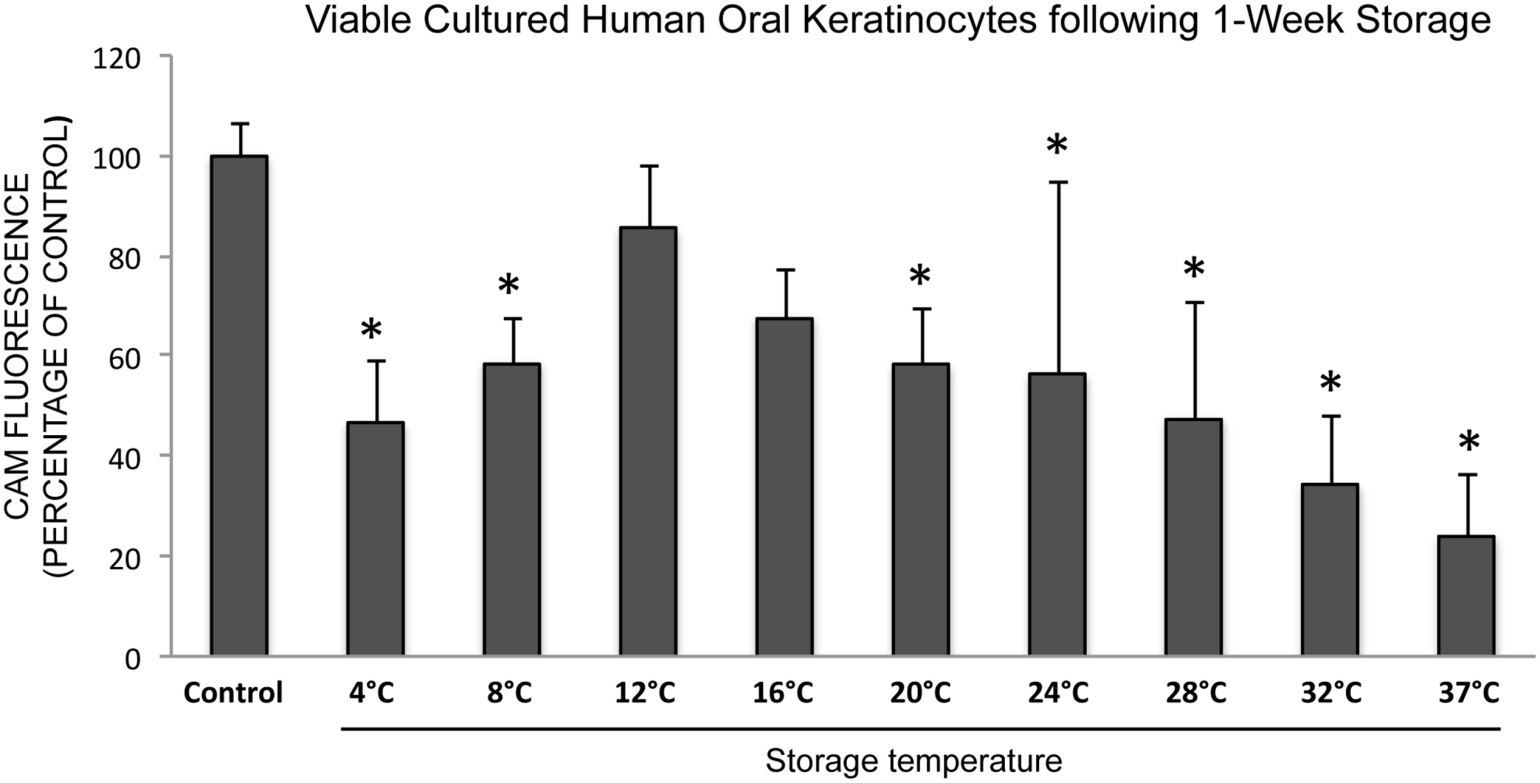
Cultured HOK were stored for seven days at nine different temperatures and viability was assessed by measuring calcein-acetoxymethyl ester (CAM) fluorescence with a microplate fluorometer. The bar chart illustrates the percentage of viable cells remaining after storage compared to control cells. (N = 5) * *P* < 0.05 compared to the control. Error bars indicate standard error of the mean.

Analysis of cell survival of stored cells using CAM showed that the number of live cells after one week of storage was significantly reduced at all storage temperatures compared to the control (*P* < 0.05), except for the 12°C and 16°C groups (Fig 2). Storage at 12°C maintained the highest number of live cells (85%±12%), while the lowest number of viable cells was detached after storage at 37°C (24%±13%), relative to the control.

### Effect of Storage Temperature on Phenotype of Stored Human Oral Keratinocyte Cultures

**Fig 3:**
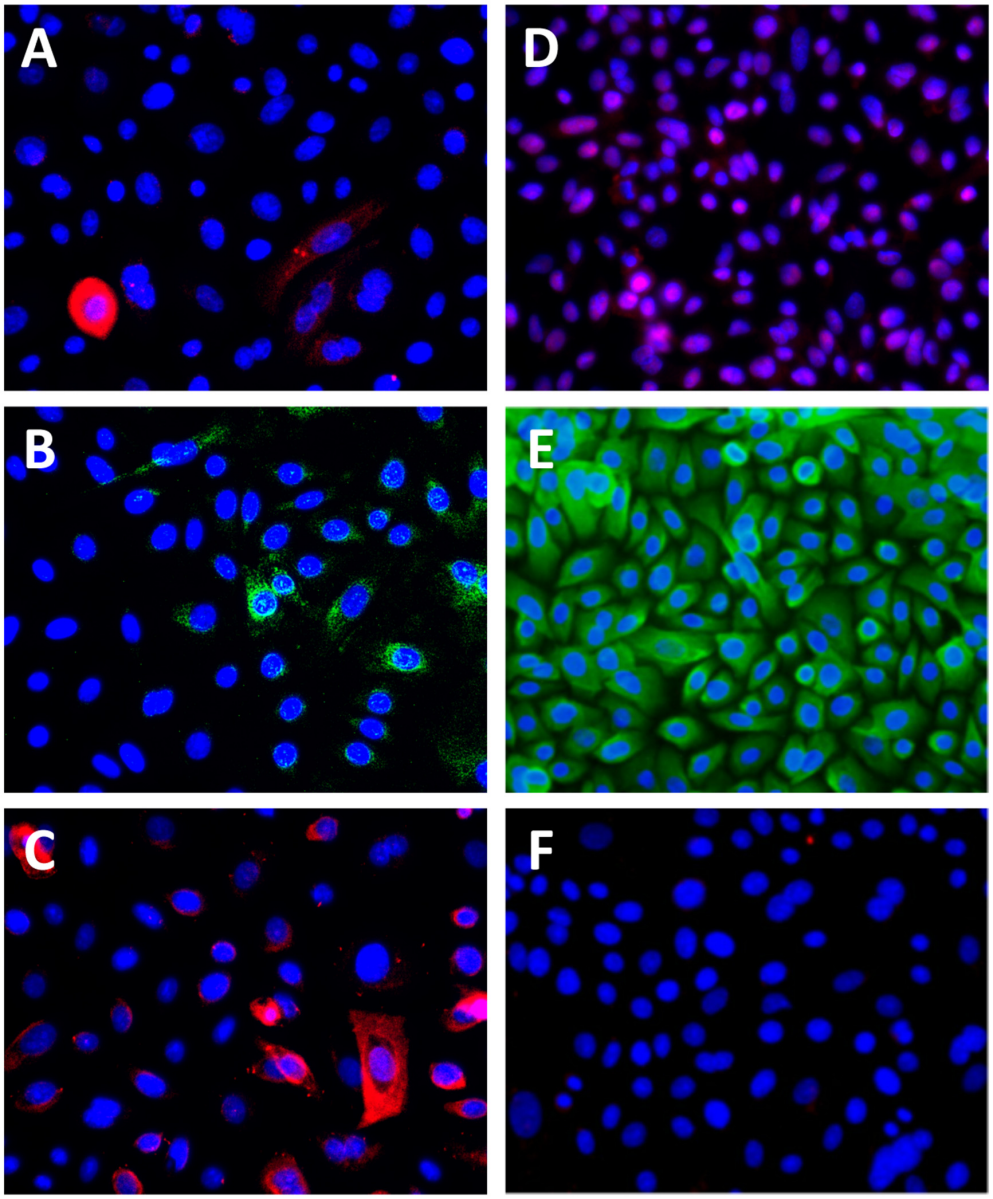
Photomicrographs showing immunostaining of (A) ABCG2 (red), (B) Bmi-1 (green), (C) C/EBPδ (red), (D) PCNA (red), (E) CK18 (green), and (F) cleaved caspase-3 (red) in HOK cultured for five days without subsequent storage (control). Photomicrographs are representative of four independent samples. Cell nuclei were counterstained with DAPI (blue). Original magnification: 200x.

HOK phenotype was analyzed in cultured HOK before and after one week of storage at nine different temperatures (Table 2). The cell membrane-associated putative stem cell marker ABCG2 [23] was maintained at a low level in all temperature groups compared to the control (Fig 3A). Bmi1, a nucleocytoplasmic putative stem cell marker involved in stem cell self-renewal [24], was detected in the control cells (Fig 3B) and conserved during storage at temperatures between 4°C and 24°C, but was decreased in the 28°C to 37°C groups (Table 2). C/EBPδ regulates self-renewal and the cell cycle in stem cells and is normally located in the cell nucleus [24]. In the cultured HOK, this marker was mostly found in the cell cytosol where it appeared unchanged in all storage groups compared to the control, except the 37°C group, where it was increased (Fig 3C) (Table 1). Nuclear expression of the proliferation marker PCNA (Fig 3D) [25] was maintained after storage at 4°C to 20°C, while it had decreased in the 24°C to 37°C groups (Table 1). CK18, present in the cytosol of HOK, (Fig 3E) [26] was similarly expressed after storage between 4°C and 24°C, compared to the control, but had decreased after storage between 28°C and 37°C (Table 1). Cleaved caspase-3, a cytoplasmic and perinuclear marker for apoptosis, (Fig 3F) increased only after storage at 37°C, compared to the control (Table 1). Collectively, phenotype was maintained in HOK cultures stored at 4°C to 20°C.

**Table 2:** List of the antibodies.

Antigen	Control	4°C	8°C	12°C	16°C	20°C	24°C	28°C	32°C	37°C
**ABCG2**	+	+	+	+	+	+	+	+	+	+
**Bmi1**	++	++	++	++	++	++	++	+	+	+
**C/EBPδ**	+++	+++	+++	+++	+++	+++	+++	+++	+++	++++
**PCNA**	++++	++++	++++	++++	++++	++++	+++	++	++	+
**CK18**	++++	++++	++++	++++	++++	++++	++++	+++	+++	+++
**Caspase 3**	+	+	+	+	+	+	+	+	+	++

The immunoreactivity was graded as 0 (undetectable), + (detectable in <1/4 of the cells), ++ (detectable in 1/4–1/2 of the cells), +++ (detectable in 1/2-3/4 of the cells) and ++++ (detectable in >3/4 of the cells). All scores were assigned at a magnification of 200x by experienced investigators.

### Effect of Storage Temperature on Cellular Metabolites in Stored Human Oral Keratinocyte Cultures

**Fig 4:**
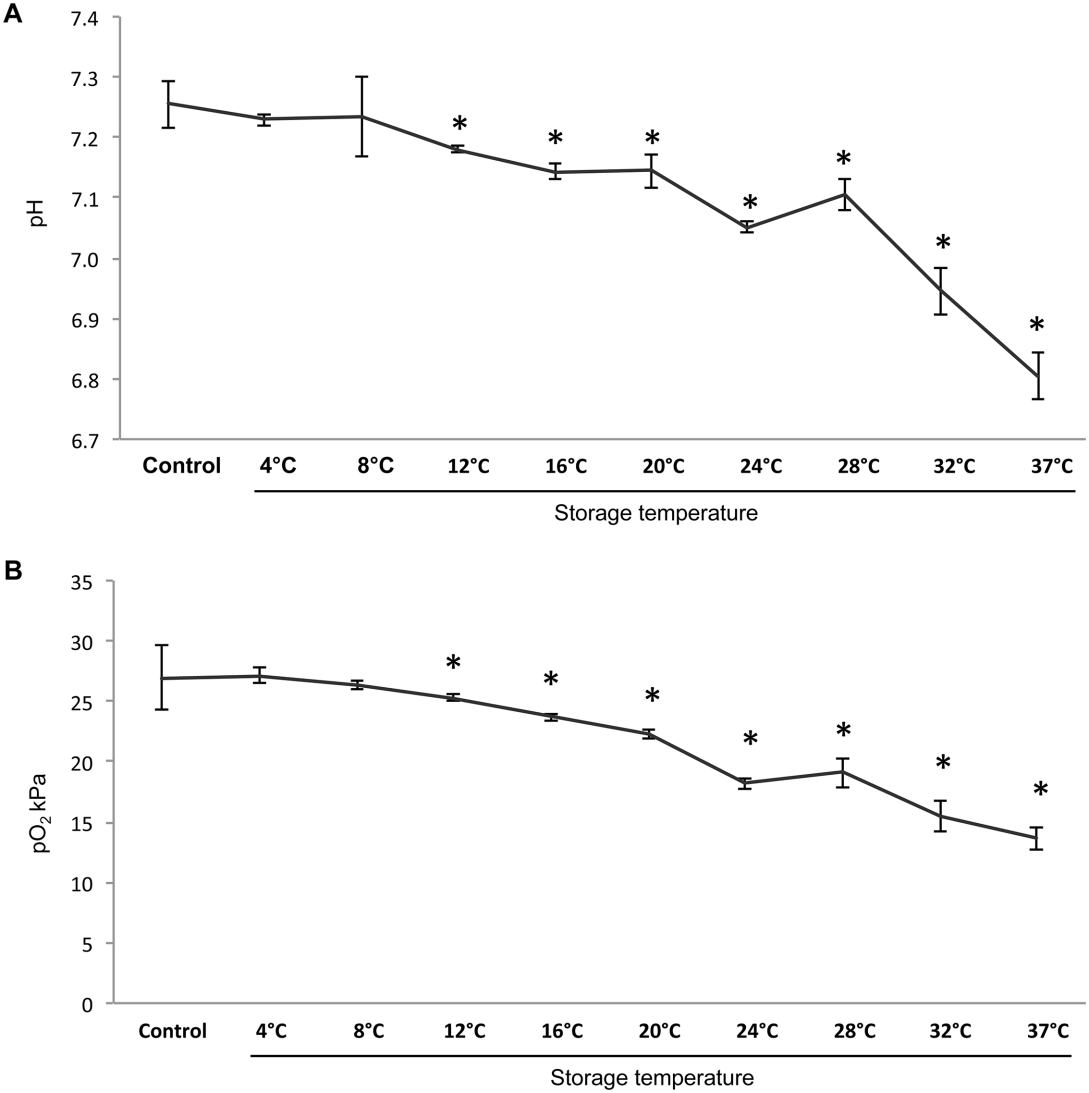
To assess the effect of storage temperature on HOK metabolism the (A) pH in the storage medium and (B) partial pressure of oxygen (pO_2_ kPa) was measured by a blood gas analyzer following seven days of storage. (N = 8 for stored samples and N = 5 for samples not subjected to storage (control)). * P < 0.05 compared to control. Error bars indicate standard error of the mean.

**Fig 5:**
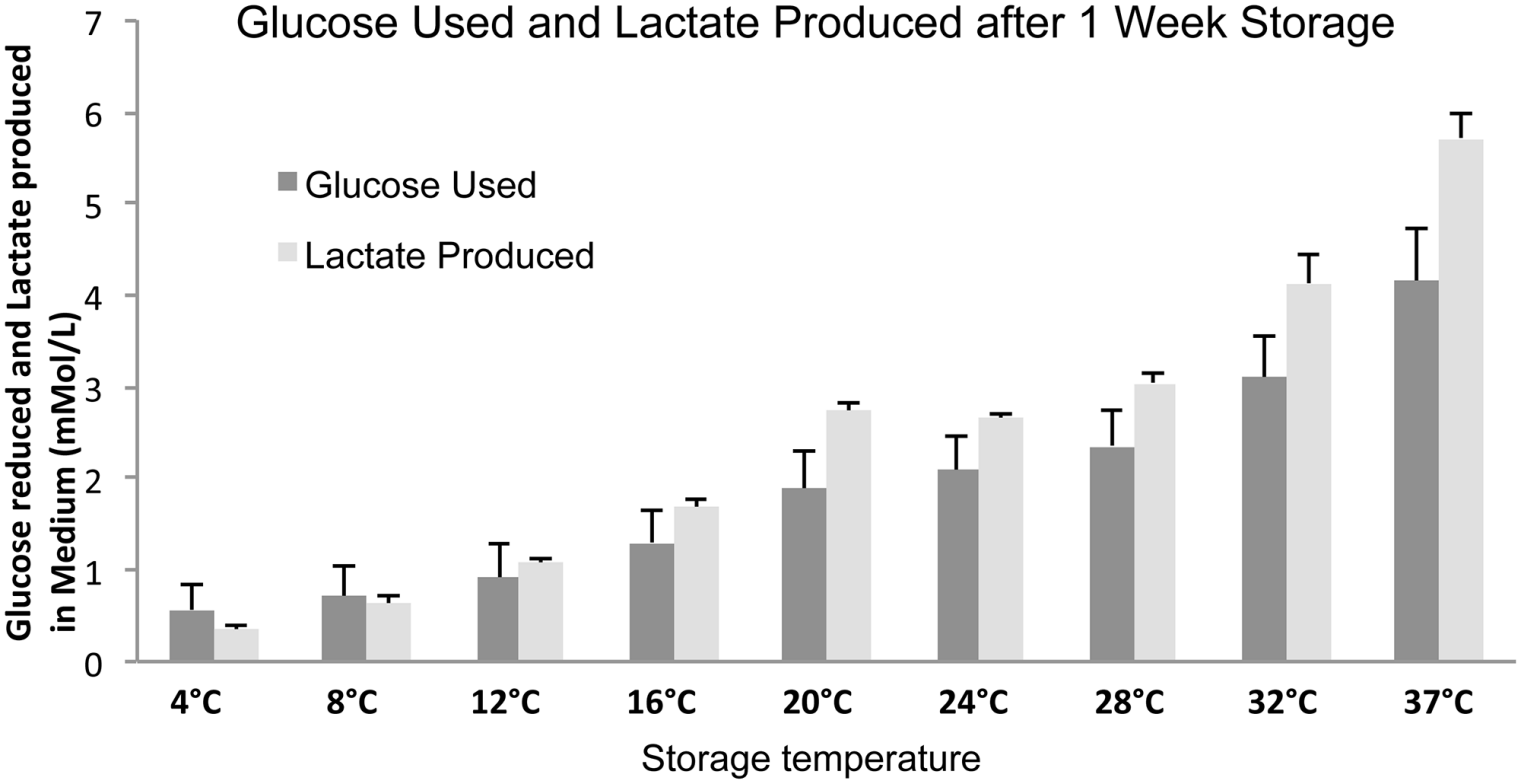
To assess the effect of storage temperature on HOK metabolism the glucose used was calculated and is presented in relation to lactate produced. Freshly prepared medium served as control (N = 8). Error bars indicate standard error of the mean.

The pH, glucose, lactate, and O_2_ were measured in the storage medium following one week of storage to investigate the effect of temperature on cell metabolism. The pH in the storage medium had decreased following one week of storage with increasing temperatures between 4°C and 37°C compared to control (*P* < 0.005) (Fig 4A). Oxygen tension also showed a steady decline from 4°C to 37°C (Fig 4B). Similar to pH, oxygen tension significantly decreased at temperatures above 12°C. Utilization of glucose by cells and concomitant production of lactate was measured subsequent to storage at 4°C to 37°C (Fig 5). Glucose used and lactate produced increased with increasing storage temperature.

### Effect of Storage Temperature on Morphology of Stored Human Oral Keratinocyte Cultures

**Fig 6:**
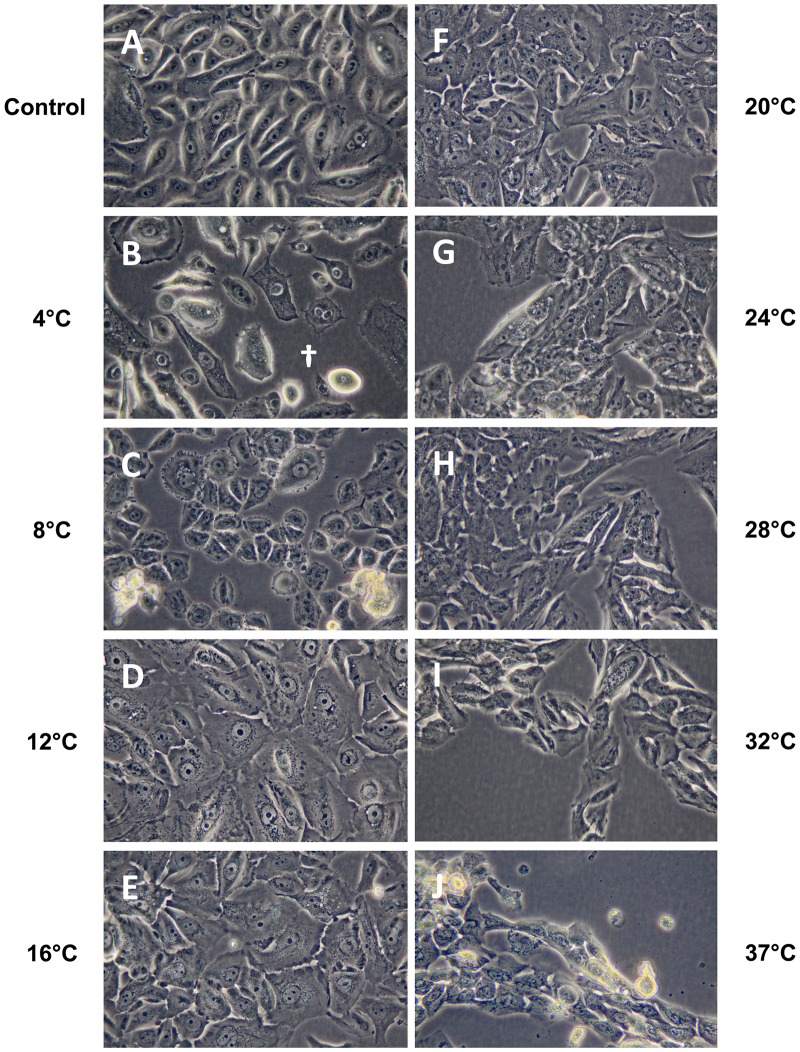
Photomicrographs of cultured HOK stored for seven days at nine temperatures were captured with an inverted light microscope with 400x magnification (A-J). Photomicrographs are representative of two independent samples. White cross (B) shows an intercellular space.

**Fig 7:**
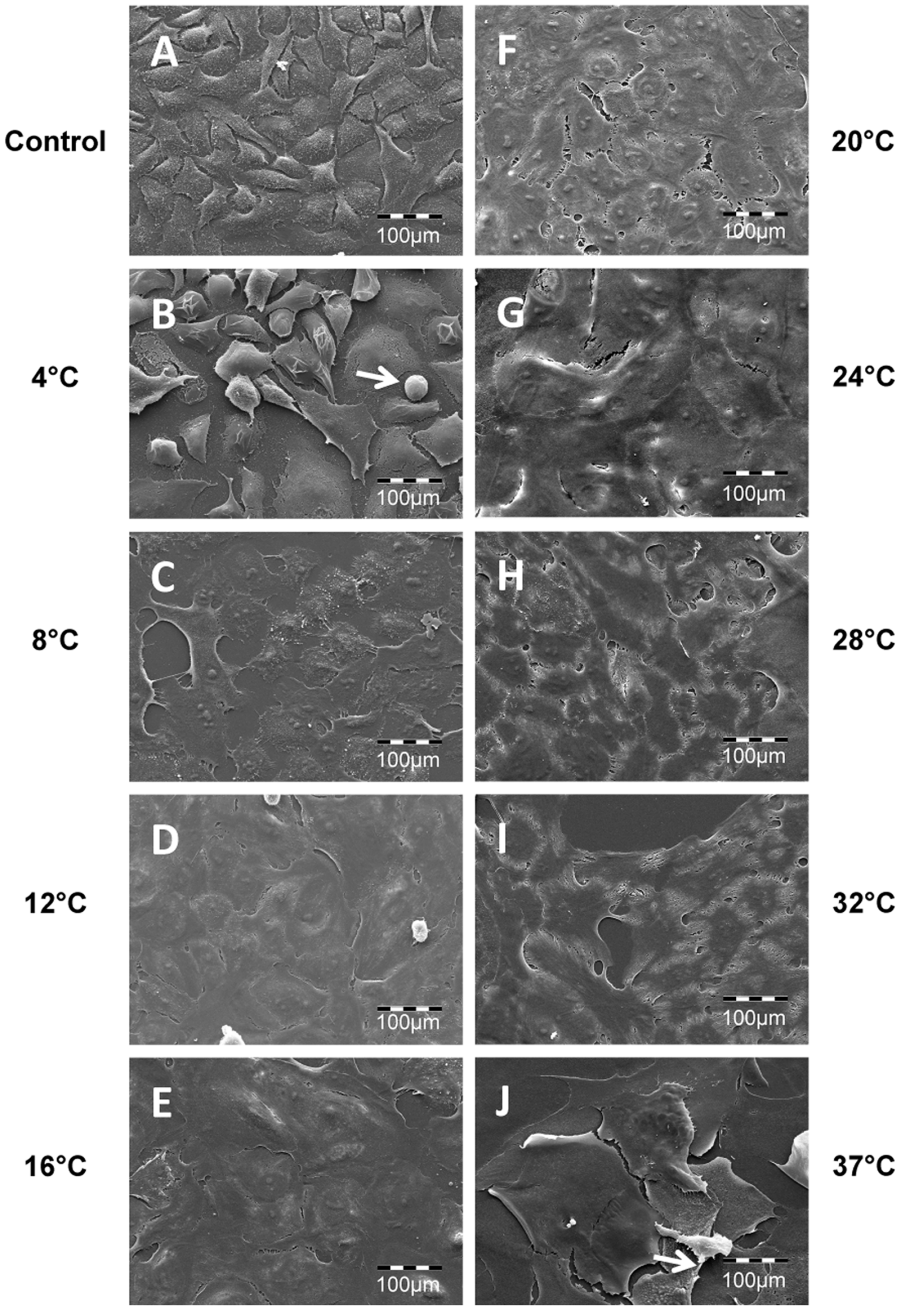
Photomicrographs of cultured HOK stored for seven days at nine temperatures were captured with a scanning electron microscope with 550x magnification (A-J). Cultured HOK cells not subjected to storage served as control. Photomicrographs are representative of two independent samples. White arrow (B) shows cell shrinkage and the white arrow in (J) shows the detachment of the cells.

**Fig 8:**
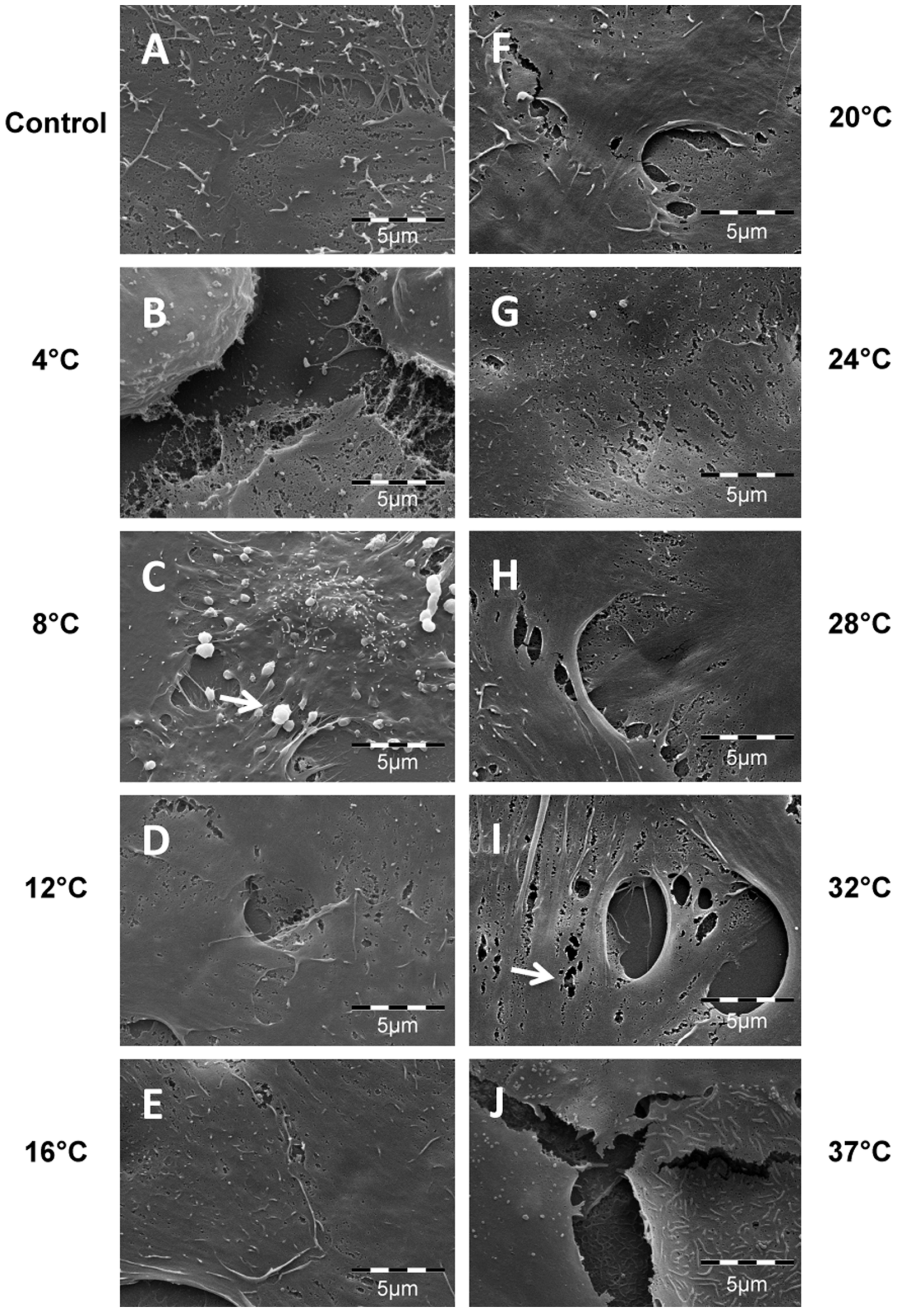
Photomicrographs of cultured HOK stored for seven days at nine temperatures were captured with a scanning electron microscope with 6000x magnification (A-J). Cultured HOK cells not subjected to storage served as control. Photomicrographs are representative of two independent samples. The white arrow in (C) shows the apoptotic body. White arrow (I) shows artifacts due to sample preparation.

Light and scanning electron microscopy were performed to examine respectively the effect of storage temperature on the overall morphology and the ultrastructure of cultured HOK. Prior to storage, the cells were generally well apposed and displayed normal epithelial morphology (Fig 6A). After storage at the lower storage temperature groups of, 4°C and 8°C, intercellular spacing was observed (Fig 6B and 6C). At higher temperature groups from 24°C to 37°C, voids due to cell loss were seen (Fig 6G–6J). Moreover, in scanning electron microscopy the lower temperature storage group showed prominent signs of apoptosis (Fig 7B). At higher temperatures morphological deformation, shrinkage, and membrane blebbing were the prominent features (Fig 8B). In contrast, the midrange temperature groups of 12°C, 16°C and 20°C resulted in the best preserved morphology.

## Discussion

The present study demonstrates a profound effect of storage temperature on cultured HOK viability, metabolism and structure, but not on phenotype. Storage at 12°C to 16°C ensured most optimal preservation of HOK cultures with maintained viability and phenotype for seven days. Although it has not been demonstrated clinically, high viability of transplanted cultured HOK is generally assumed to be, advantageous for clinical success in the treatment of LSCD. In line with our finding that storage at 4°C yielded a viability of 46%, Lee *et al*. (2005) showed that viability of HOK stored in suspension in DMEM deteriorates by ~60% after three days [27]. By adding human serum albumin to the storage medium, the authors increased the viability to ~80% at both 4°C and at room temperature. Their short storage period may have masked any temperature associated effects on viability.

The impact of storage temperature on viability of cultured cells has been investigated in other cell types. Raeder *et al*. [28] reported that storage of cultured HLEC at 23°C was superior to storage at both 5°C and 31°C. Eidet *et al*. [20] demonstrated that primary cultures of human conjunctival epithelial cells on amniotic membranes maintained viability after seven days of storage at 23°C. When cultured conjunctival epithelial cells were cultured on plastic rather than amniotic membrane, the viability was most favorable at 12°C [18]. Recently, Pasovic *et al*. showed that temperatures between 12°C and 20°C were best for maintaining retinal pigment epithelial cell viability. Thus, our results showing optimal viability after storage of oral epithelium at 12°C and 16°C are in agreement with previous studies on storage of cultured cells.

Characterization of HOK cells in the present study showed that cell storage at all temperatures maintained the phenotype. There are no studies reporting immunostaining of cultured HOK following storage. However, the levels of the putative stem cell markers ABCG2, Bmi-1 and C/EBPδ described in our results are in agreement with most of our storage studies on other cultured epithelial cells. In these studies cells maintained expression of undifferentiated markers following storage [17, 19, 20].

PCNA, an endogenous nuclear protein, has been used to identify replicating cells. Expression of PCNA occurs during the late G1 phase, reaches its peak during S phase and continues into mitosis [29]. Maintenance of PCNA at a level similar to the control was only detected following storage between 4°C and 20°C. This accords with a study by Rieder and Cole, suggesting that progression through mitosis is temperature sensitive and the cell cycle is prolonged at 20°C and below [30]. The best storage temperatures for preserving viability during one week of storage overlapped to some degree with the temperatures that demonstrated the highest percentage of PCNA positive cells (e.g. 12°C to 16°C), suggesting that conserved proliferative function could be one important criterion for successful cell storage.

CK18, a commonly used marker for HOK, was used to show the differentiation profile throughout the temperature range after one week of storage. The consistent expression of CK18 suggests that the cells did not undergo differentiation at any of the temperatures. In our study, cleaved caspase-3 expression was unchanged after storage despite great variations in morphology of the cultured cells at different storage temperatures. However, the number of calcein positive live cells after storage had dropped significantly in most of the groups, compared to the control. The low percentage of cleaved caspase-3 positive cells can be explained by the dead cells’ tendency off dead cells to detach and be washed away during rinsing prior to immunostaining. The low number of cleaved caspase-3 positive cells after storage at 12°C and 16°C is in agreement with that found following storage of cultured human conjunctival and retinal pigment epithelial cells [18, 19].

To investigate the relationship between metabolism and viability during storage at the different temperatures, glucose and lactate measurements taken from the storage media were compared with viability. Glucose consumption progressively increased in a temperature responsive manner following an expected increase in metabolic rate with temperature [31]. Low energy production, inferred by metabolic values at 4°C and 8°C, was particularly aligned with low viability at these temperatures. However, the high viability at 12°C and 16°C did not lead to increased glucose consumption at these two temperatures. Glucose consumption and lactate accumulation were strongly correlated with a negative trend in viability seen between 12°C and 37°C (*r* = -0.784 and -0.700, respectively; *P* < 0.001 in both cases). It has been shown previously that progressively higher concentrations of lactate accumulate in the medium following storage of whole pig corneas at 4°C, 21°C and 31°C for six days [32]. In accordance with our results, the study by Reim *et al*. showed that metabolism was severely repressed at 4°C, as indicated by low lactate accumulation and ATP levels. Corneas stored at 21°C and 31°C demonstrated comparatively higher metabolic activity, and constant lactate levels throughout the six day period at 21°C suggested a balance between the two major metabolic pathways, glycolysis and oxidative phosphorylation. The calculated lactose/glucose ratio in our study was ~1 at 4°C and 8°C, and fluctuated around 1.3 between 12°C and 37°C, suggesting that the glycolysis accounted for at least partial energy production at all temperatures [33].

While the storage media became slightly more acidic with increasing temperature, pH remained within physiological range. It was, therefore, surprising to see that viability progressively decreased with temperature whereas energy production appeared to increase. Energy production would normally be associated with an increase in number of viable cells. Use of the glycolytic pathway as seen here could represent an adjustment to avoid excessive production of damaging reactive oxygen species generated through the oxidative phosphorylation pathway [34].

After one week of storage, the cells stored at both 4°C and 8°C demonstrated increased intercellular space. Subsequent to storage at 4°C, shrinkage of cytoplasm was observed. The 8°C storage group showed membrane blebbing. These findings are in line with previous work demonstrating that cold storage induces both apoptosis and necrosis [35]. Cells stored between 24°C and 37°C also occasionally displayed morphology associated with apoptosis. Several empty areas were seen within the cell sheet due to cell detachment and cell shrinkage, correlating with the reduction in viability values measured at these temperatures [36]. These morphological findings are in accordance with a previous study on storage of cultured HLEC that demonstrated pronounced cell detachment following storage at 31°C [22]. The morphology presented in the present study was generally in agreement with the viability data, showing best cell preservation at 12°C and 16°C.

In conclusion, the current study demonstrates that cultured HOK are best preserved at a temperature range between 12°C and 16°C. Importantly, the storage method described in the present study may be applicable for other cell types and tissues; thus its significance may extend beyond HOK and the field of ophthalmology.
